# Nitrosative Stress-Induced Disruption of Baroreflex Neural Circuits in a Rat Model of Hepatic Encephalopathy: A DTI Study

**DOI:** 10.1038/srep40111

**Published:** 2017-01-12

**Authors:** Ching-Yi Tsai, Chia-Hao Su, Julie Y. H. Chan, Samuel H. H. Chan

**Affiliations:** 1 Institute for Translational Research in Biomedicine, Kaohsiung Chang Gung Memorial Hospital, Kaohsiung, Taiwan, Republic of China

## Abstract

The onset of hepatic encephalopathy (HE) in liver failure is associated with high mortality; the underlying mechanism is undecided. Here we report that in an acute liver failure model employing intraperitoneal administration of thioacetamide in Sprague-Dawley rats, diffusion weighted imaging revealed a progressive reduction in apparent diffusion coefficient in the brain stem. Diffusion tensor imaging further showed that the connectivity between nucleus tractus solitarii (NTS), the terminal site of baroreceptor afferents in brain stem and rostral ventrolateral medulla (RVLM), the origin of sympathetic innervation of blood vessels, was progressively disrupted until its disappearance, coincidental with the irreversible cessation of baroreflex-mediated sympathetic vasomotor tone signifying clinically the occurrence of brain death. In addition, superoxide, nitric oxide, peroxynitrite and ammonia levels in the NTS or RVLM were elevated, alongside swelling of astroctytes. A scavenger of peroxynitrite, but not an antioxidant, delivered intracisternally reversed all these events. We conclude that nitrosative stress because of augmented peroxynitrite related to accumulation of ammonia and swelling of astrocytes in the NTS or RVLM, leading to cytotoxic edema in the brain stem and severance of the NTS-RVLM connectivity, underpins the defunct baroreflex-mediated sympathetic vasomotor tone that accounts for the high mortality associated with HE.

Hepatic encephalopathy (HE) reflects a serious and progressive disorder with a wide spectrum of neuropsychiatric abnormalities and motor disturbances that accompanies the acute onset of severe hepatic dysfunctions^1^,^2^,^3^,^4^. It is most commonly seen in patients with advanced liver cirrhosis secondary to significant portosystemic shunting and severe hepatocellular dysfunction. In acute hepatic failure, the onset of encephalopathy is an indication for liver transplantation^1^, without which is associated with 50–90% mortality^5^. Mechanisms that have been suggested to underpin HE include brain edema^6^, oxidative stress^7^, inflammation^6^,^8^, mitochondrial dysfunction^9^, activation of astrocytes or microglia^6^,^10^, increase in ammonia^8^ and enhanced inhibitory neurotransmission in brain^4^. However, none of these mechanisms specifically addresses the high mortality aspect of HE.

Under physiological conditions, the baroreflex-mediated sympathetic vasomotor tone and cardiac vagal baroreflex are responsible respectively for the maintenance of blood pressure (BP) and heart rate (HR)^11^. Nonetheless, under pathological conditions, our laboratory has demonstrated in comatose patients that irreversible loss of baroreflex-mediated sympathetic vasomotor tone is a hallmark for brain death^12^,^13^,^14^,^15^. We further identified that nitrosative stress in key nuclei of the neural circuit is the culprit for the defunct baroreflex^16^ in animal models of brain death^16^,^17^,^18^,^19^,^20^,^21^,^22^,^23^,^24^,^25^. Intriguingly, patients with alcoholic cirrhosis exhibit reduced baroreflex sensitivity^26^, and the degree of decreased HR variability is related to the severity of HE in patients with cirrhosis^27^. Together, these observations implicated a causal role for defunct baroreflex-mediated sympathetic vasomotor tone in high mortality associated with HE.

Several studies^28^,^29^,^30^ applied magnetic resonance imaging (MRI) coupled with diffusion weighted imaging (DWI) or diffusion tensor imaging (DTI) to investigate the intracellular or extracellular distribution and mobility of water in forebrain structures of patients with fulminant hepatic failure or in rat model of acute liver failure. The common conclusion is that cytotoxic cell swelling underpins cerebral edema, and that ammonia and astrocytes are intimately involved. We noted that MRI/DWI/DTI have not been applied extensively to small brain areas such as the brain stem, let alone in mechanistic investigation of the high mortality associated with HE. At the same time, our laboratory reported recently that when coupled with relevant cardiovascular phenotypes, MRI/DTI of the baroreflex neural circuits in the brain stem can be an effective investigative tool for functional evaluations of baroreflex activities^31^. Following those leads, the present study employed MRI/DWI/DTI of the rat brain stem to evaluate the guiding hypothesis that impairment of baroreflex-mediated sympathetic vasomotor tone because of nitrosative stress in the nucleus tractus solitarii (NTS), the terminal site of the primary baroreceptor afferents^32^ or the rostral ventrolateral medulla (RVLM), which mediates reflex adjustment of sympathetic outflow to the blood vessels^33^, underpins the high mortality associated with HE. Based on a clinically-relevant rat model of acute liver injury, this hypothesis was validated.

## Results

### Rat model of hepatic encephalopathy

Our first series of experiments established a rat model that is compatible with the clinical observations from patients with HE. Sprague-Dawley rats that received daily intraperitoneal injection of thioacetamide (TAA) at doses of 300, 400 or 500 mg kg^−1^ for 3 consecutive days exhibited a significant and dose-related increase in serum levels of aspartate aminotransferase (AST), alanine aminotransferase (ALT), total bilirubin (T-Bilirubin) or blood level of ammonia, and decrease in serum level of albumin (Table S1). Those biochemical markers of liver damage were corroborated by liver necrosis and neutrophil infiltration around the centrilobular vein in TAA-treated rats (Fig. S1). Based on the classifications by the West Haven scale^4^,^34^, rats also displayed dose-related increase in severity of HE (Table S2). This was reflected by the progression from mild lethargy (grade 1), to decreased motor activity, poor gesture control and diminished pain perception (grade 2), severe ataxia, with no spontaneous righting reflex (grade 3) to disappearance of righting reflex and no reaction to pain stimuli (grade 4)^35^. In addition, there was a dose-related increase in mortality rate when determined on Day 4 after TAA treatment (Table S3). To allow for an appropriate time-window to carry out our MRI/DWI/DTI, physiological and biochemical studies, we routinely followed, unless specifically mentioned, the responses to a dose of TAA at 300 mg kg^−1^ for 4 days in subsequent experiments.

### The progressive changes in cardiac vagal baroreflex and baroreflex-mediated sympathetic vasomotor tone exhibited differential time-courses during experimental HE

**Figure 1:**
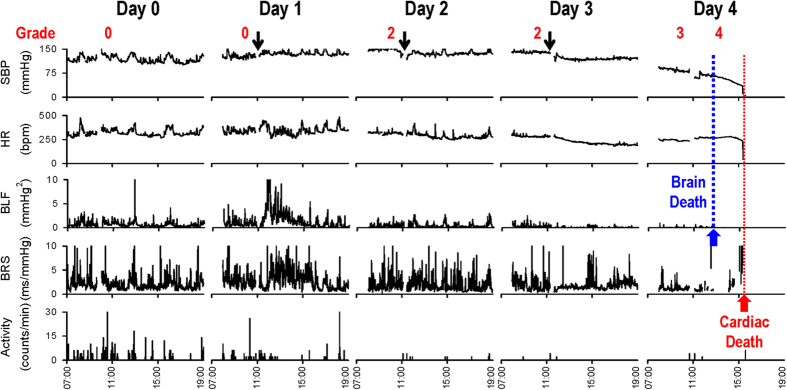
Rat model of hepatic encephalopathy (HE) showing the progression from normal to dysfunctional and defunct baroreflex. Illustrative example of daily intraperitoneal administration of thioacetamide (TAA) at a dose of 500 mg kg^−1^ for 3 consecutive days (arrows) on systolic blood pressure (SBP), heart rate (HR), power of low-frequency component of SBP spectrum (BLF), an index for baroreflex-mediated sympathetic vasomotor tone, baroreflex sensitivity (BRS), an index for cardiac vagal baroreflex, and physical activity as measured by radiotelemetry in conscious rats. Numbers at the top were grading of the severity of HE taken immediately before TAA injection. The time point when brain death or cardiac death occurs is denoted respectively by blue arrow and blue gross dotted line and red arrow and red fine dotted line.

Results from radiotelemetry coupled with on-line and real-time spectral analysis of HR and systolic BP (SBP) signals revealed several intriguing observations (Fig. 1) in our rat model of HE. First, the validity of our grading system was confirmed by the progressive decrease in physical activities beginning on Day 2. Second, superimposed on a trend of slow reduction, HR was essentially maintained until the abrupt occurrence of asystole signifying cardiac death (red arrow and red fine dotted line). As reflected by the persistent presence of spontaneous baroreflex sensitivity (BRS)^31^, there was sustained cardiac vagal baroreflex until shortly before the abrupt occurrence of cardiac arrest. Of note is that BRS was actually discernibly enhanced to maintain HR after the first TAA injection on Day 1. Third, drastic reduction in SBP began to appear on Day 3, and at an accelerated rate on Day 4. More intriguingly, the power density of the low-power frequency (BLF) component in the SBP spectrum (0.25–0.8 Hz) was augmented after the first injection of TAA on Day 1 to maintain SBP. This index of baroreflex-mediated sympathetic vasomotor tone^36^ began to decrease on Day 2, and reached zero on Day 4 (blue arrow and blue gross dotted line) coincidental with a behavioral grade of 4. Clinically, this signifies brain death in comatose patients despite the presence of reasonable SBP and maintained HR^12^,^13^,^14^,^15^.

### Edema formation in brain stem

**Figure 2:**
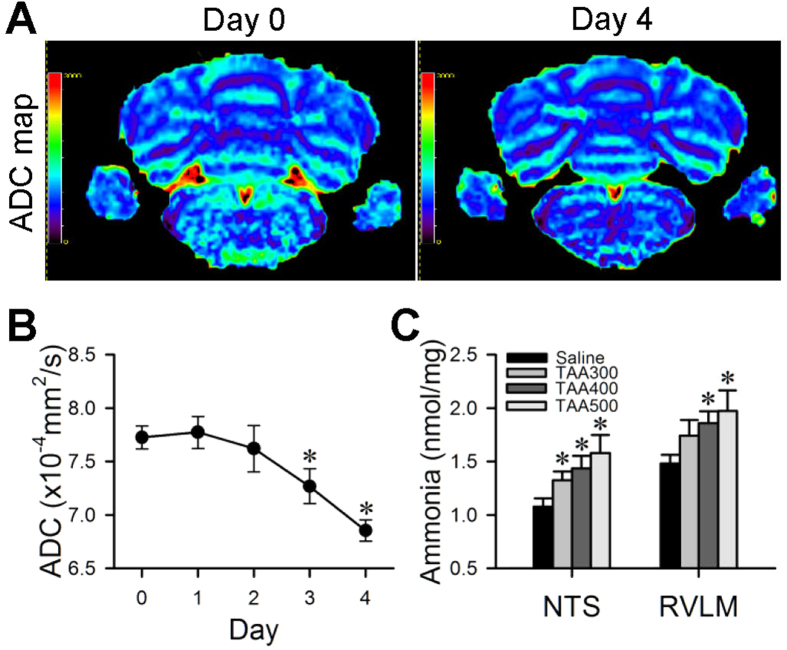
Presence of cytotoxic edema in brain stem and augmented tissue ammonia in the NTS or RVLM during experimental HE. Illustrative example of apparent diffusion coefficient (ADC) maps of the brain stem obtained by DWI **(A)**; or the temporal changes in ADC, a quantitative index for water mobility **(B)** before (Day 0), during (Days 1 to 3) and after (Day 4) TAA administration. **(C)** Dose-related effects of TAA on tissue levels of ammonia in the NTS or RVLM detected on Day 4. Values in **(B)** and **(C)** are mean ± SEM, n = 5 animals in each group. *P < 0.05 versus baseline control (Day 0) group in **(B)** or saline group in **(C)** in the post hoc Dunnett **(B)** or Scheffé **(C)** multiple-range analysis. Scale bars = 0 to 2000 arbitrary units.

As indicated by an overall reduction in apparent diffusion coefficient (ADC) (Fig. 2A), formation of cytotoxic edema^28^ in the brain stem occurred during experimental HE. Further analysis showed that significant decrease in the ADC of the brain stem (Fig. 2B) began on Day 3 after the first injection of TAA, followed by further decline on Day 4. There was also a significant reduction in axial diffusivity (λ_∥_) in the brain stem on Days 3 and 4 after TAA treatment (Table S4), although changes in radial diffusivity (λ_**⊥**_) were insignificant. Of note is that TAA also elicited a dose-dependent increase in tissue level of ammonia in the NTS and RVLM (Fig. 2C).

### Augmented number and size of astrocytes in NTS and RVLM

**Figure 3:**
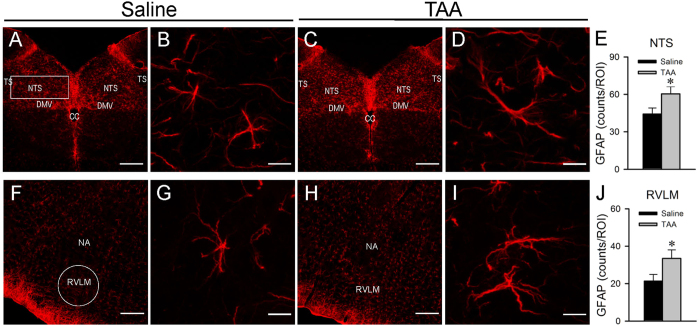
Augmented number and size of astrocytes in the NTS or RVLM during experimental HE. Representative laser scanning confocal microscopic images showing low- **(A,C,F,H)** and high-power **(B,D,G,I)** views of cells in the dorsal (**A–D**) or ventral (**F–I**) medulla oblongata that were immunoreactive to the astrocyte marker GFAP in rats treated with saline or TAA. **(E,J)** Quantitative analysis of GFAP immunoreactivity using ImageJ in region of interest denoted in (**A,F**). Values are mean ± SEM, n = 5 animals in each group. *P < 0.05 versus saline group in Student’s t-test. Scale bars, 200 μm in **(A,C,F,H)** and 10 μm in **(B,D,G,I)**. CC = central canal; DMV = dorsal motor nucleus of vagus nerve; NA = nucleus ambiguus; NTS = nucleus tractus solitarii; RVLM = rostral ventrolateral medulla; TS = tractus solitarii.

Immunofluorescence staining showed that the number of glial fibrillary acidic protein (GFAP) immunoreactive astrocytes in the NTS (Fig. 3A–E) and RVLM (Fig. 3F–J) exhibited a significant increase during experimental HE. In addition, many of these astrocytes also underwent swelling of their cell body and processes (Fig. 3B,D,G,I).

### Differential disruption of neural circuits for cardiac vagal baroreflex and baroreflex-mediated sympathetic vasomotor tone during experimental HE

**Figure 4:**
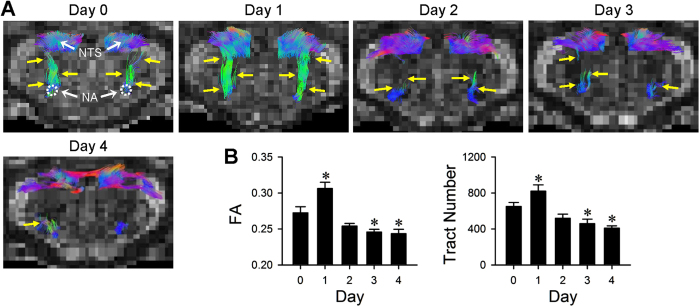
Disrupted connectivity between the NTS and NA during experimental HE. Illustrative example of imaging connectivity (yellow arrows) between the NTS and NA in rats by DTI **(A)**; or the associated temporal changes in fractional anisotropy (FA) or number of tracts connecting the NTS and NA, quantitative indices for the magnitude of connectivity in DTI analysis **(B)** before (Day 0), during (Days 1 to 3) and after (Day 4) TAA administration. Values in **(B)** are mean ± SEM, n = 7 animals. *P < 0.05 versus baseline control (Day 0) group in the post hoc Dunnett multiple-range analysis. Note that in this and Figs 5 and 8, representative colors for tractography in color-encoded FA maps are: blue, caudal-rostral; red, left-right; and green, dorsal-ventral. Note also that a reduction denotes disruption of connectivity.

**Figure 5:**
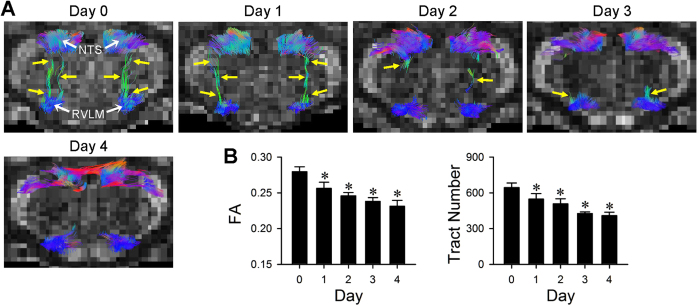
From disrupted to severed connectivity between the NTS and RVLM during experimental HE. Illustrative example of imaging connectivity (yellow arrows) between the NTS and RVLM in rats by DTI **(A)**; or the associated temporal changes in FA or number of tracts connecting the NTS and RVLM, quantitative indices for the magnitude of connectivity in DTI analysis **(B)** before (Day 0), during (Days 1 to 3) and after (Day 4) TAA administration. Values in **(B)**, are mean ± SEM, n = 7 animals. *P < 0.05 versus baseline control (Day 0) group in the post hoc Dunnett multiple-range analysis.

Tractographic analysis based on DTI of the brain stem further revealed that the temporal changes in connectivity between the NTS and nucleus ambiguus (NA) (Fig. 4A), the origin of vagal innervation of the heart, during the 4-day course of experimental HE manifested a trend that resembled that of the spontaneous BRS (Fig. 1). Similar to the enhanced BRS, this NTS-NA connectivity was also discernibly augmented after the first TAA injection on Day 1. Again, despite a trend of partial disruption, the connectivity between the NTS and NA was sustained until shortly before asystole took place during experimental HE (Fig. 4A). Likewise, the temporal changes in connectivity between the NTS and RVLM (Fig. 5A) during the 4-day course of experimental HE manifested a trend that resembled that of the power density of the BLF component of SBP spectrum (Fig. 1). The connectivity between the NTS and RVLM (Fig. 5A) exhibited an appreciable disruption after the first injection of TAA on Day 1, to be followed by further reduction that began on Day 2. The NTS-RVLM connectivity was completely eliminated, coincidental with a behavioral grade of 4 and loss of BLF power on Day 4. Quantification by fractional anisotropy (FA) and number of fiber tracts revealed comparable results (Figs 4B and 5B). As a negative control, tractographic analyses of the bilateral pyramidal tracts (Fig. S2), of which does not play a role in baroreflex regulation of the heart or blood vessels, showed consistent DTI images and FA values or number of fiber tracts only in the caudal-rostral direction during the course of experimental HE.

### Nitrosative stress in NTS and RVLM underpins impairment of baroreflex and disruption of NTS-NA or NTS-RVLM connectivity

**Figure 6:**
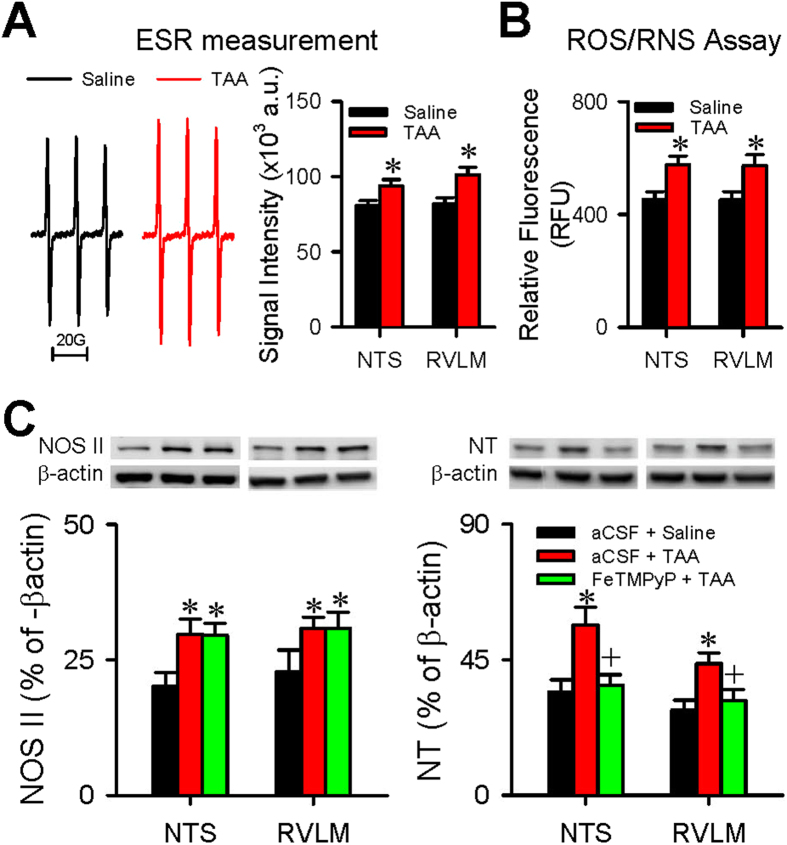
Nitrosative stress in the NTS or RVLM during experimental HE. Effects of TAA treatment on ROS/RNS production in the NTS or RVLM as detected by electron spin resonance (ESR) **(A)** or by an assay kit **(B)**. Values are mean ± SEM, n = 4-animals. *P < 0.05 versus saline in the Student’s *t*-test. **(C)** Changes in protein level of nitric oxide synthase II (NOS II) or nitrotyrosine (NT), an experimental index for peroxynitrite, showing the effects of intracisternal infusion of artificial cerebrospinal fluid (aCSF) or FeTMPyP (100 pmol μl^−1^ h^−1^), an active peroxynitrite decomposition catalyst, before (Day 0) or after (Day 4) TAA administration. Values are mean ± SEM, n = 5–7 animals. *P < 0.05 versus aCSF + saline group; and ^+^P < 0.05 versus aCSF + TAA group on Day 4 in the post hoc Scheffé multiple-range analysis. The full-length western blots for the cropped blots presented in (**C**) are included in the Supplementary Information file.

**Figure 7:**
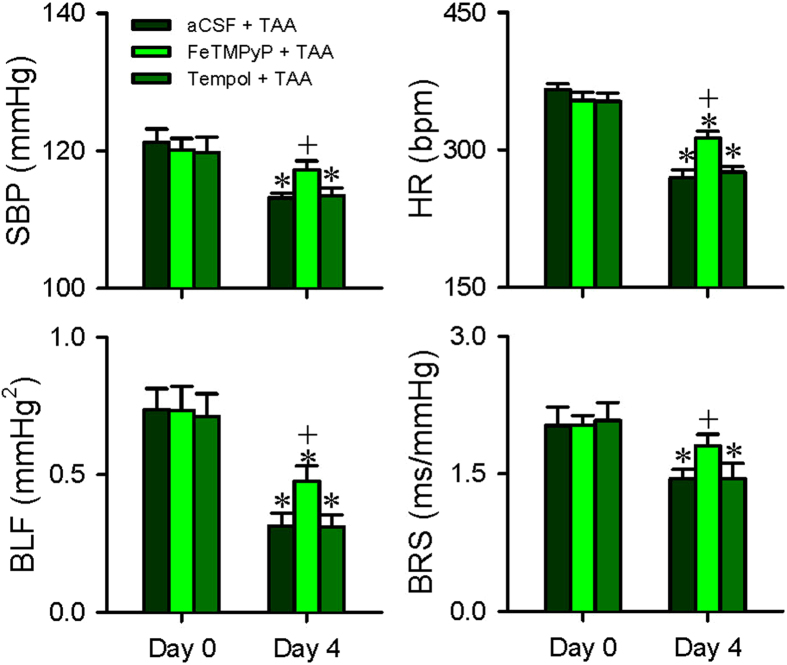
Nitrosative stress in the NTS or RVLM underlies the impaired baroreflex during experimental HE. Changes in SBP, HR, BLF power or BRS showing the effects of intracisternal infusion of aCSF, FeTMPyP (100 pmol μl^−1^ h^−1^), an active peroxynitrite decomposition catalyst, or tempol (4 nmol μl^−1^ h^−1^), an antioxidant, before (Day 0) or after (Day 4) TAA administration. Values are mean ± SEM, n = 5–7 animals. *P < 0.05 versus corresponding baseline (Day 0) group in the post hoc Dunnett multiple-range analysis; and ^+^P < 0.05 versus aCSF + TAA group on Day 4 in the post hoc Scheffé multiple-range analysis.

**Figure 8:**
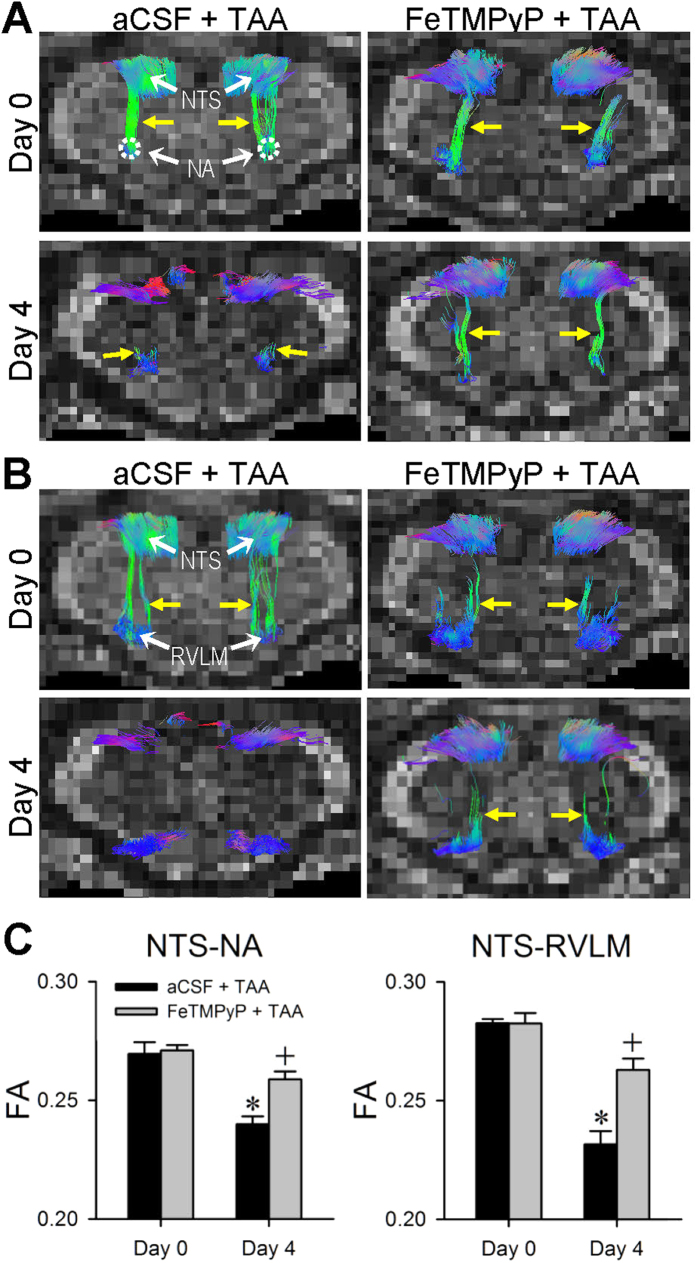
Nitrosative stress in the NTS underlies the disrupted NTA-NA or NTS-RVLM connectivity during experimental HE. Illustrative examples of imaging connectivity (yellow arrows) between the NTS and NA **(A)** or the NTS and RVLM **(B)** in rats by DTI or the associated temporal changes in FA **(C)** showing the effects of intracisternal infusion of aCSF or FeTMPyP (100 pmol μl^−1^ h^−1^) before (Day 0) or after (Day 4) TAA administration. Values in **(C)** are mean ± SEM, n = 5–7 animals. *P < 0.05 versus corresponding baseline (Day 0) group in the post hoc Dunnett multiple-range analysis; and ^+^P < 0.05 versus aCSF + TAA group on Day 4 in the post hoc Scheffé multiple-range analysis.

Our last series of experiments evaluated whether nitrosative stress in the NTS and RVLM is causally related to the impaired baroreflex and disrupted connectivity between the NTS and NA or RVLM in experimental HE. Results from both electron spin resonance (Fig. 6A) and biochemical assay (Fig. 6B) showed a significant increase in reactive oxygen species (ROS)/reactive nitrogen species (RNS) in the NTS and RVLM on Day 4 after the first TAA administration. Compared with control animals that received artificial cerebrospinal fluid (aCSF) and saline, Western blot analysis (Fig. 6C) further showed that TAA treatment augmented the expression of nitric oxide synthase II (NOS II) and nitrotyrosine, an experimental index for peroxynitrite, in both medullary sites. The augmentation of peroxynitrite in the NTS and RVLM, but not NOS II, was significantly blunted in rats that received intracisternal infusion of 5,10,15,20-tetrakis-(N-methyl-4′-pyridyl)-porphyrinato iron (III) (FeTMPyP; 100 pmol μl^−1^ h^−1^) (Fig. 6C) during experimental HE. Intracisternal infusion of this active peroxynitrite decomposition catalyst also protected against the significant reduction in SBP, HR, BLF power and BRS (Fig. 7), although pretreatment with an antioxidant, tempol (4 nmol μl^−1^ h^−1^) was ineffective. Intriguingly, pretreatment with FeTMPyP similarly reversed the disrupted connectivity between NTS and NA (Fig. 8A) or NTS and RVLM (Fig. 8B), and antagonized the corresponding reduction in FA (Fig. 8C), when examined on Day 4 after TAA treatment.

## Discussion

Using MRI/DWI/DTI as a research tool, in conjunction with physiological, biochemical and immunofluorescence experiments, the present study offers several interesting mechanistic insights on the high mortality associated with HE. Our results showed that nitrosative stress, in the form of upregulated peroxynitrite at the NTS or RVLM, is manifested in our rat model of HE. Our tractographic and physiological analyses further demonstrated that this induced nitrosative stress in the NTS or RVLM is causally related to the severance of the connectivity between the NTS and RVLM, which leads to defunct baroreflex-mediated regulation of vasomotor tone despite reasonable SBP, the hallmark of brain death. At the same time, the reduced but maintained connectivity between the NTS and NA during HE results only in dysfunctional cardiac vagal baroreflex that sustains HR until the occurrence of cardiac death. Finally, our DWI, biochemical and immunofluorescence results suggested that the above cascade of events may be related to the production of ammonia and increase in number and size of astrocytes in the NTS and RVLM, which lead to cytotoxic edema in the brain stem.

Whereas tractographic studies on forebrain structures have been published extensively, DTI evaluations have rarely been applied to small brain areas such as the brain stem. Our laboratory^31^ reported recently that when coupled with relevant physiological phenotypes, DTI can be an effective investigative tool for functional evaluations of baroreflex activities. Following this lead, the present study demonstrated the feasibility of applying tractographic analysis of the rat brain stem, in conjunction with functional assessment of both cardiac vagal baroreflex and baroreflex-mediated sympathetic vasomotor tone, for mechanistic evaluation of the high mortality associated with HE. The fundamental premise of our experimental design was based on the notion that DTI uses diffusion of water molecules as the probe to measure diffusion anisotropy and fiber orientation, and tractographic analysis is an investigative tool to determine brain connectivity. Since the passage of action potentials along the axon will create prominent anisotropy, we reasoned that a decrease in FA can be taken to infer reduction or cessation of impulse traffic between two brain structures, which we termed disruption or severance of connectivity. The specificity of our finding is confirmed by the lack of discernible changes in the DTI images of the bilateral pyramidal tracts and the associated FA and tract numbers before and after the induction of experimental HE.

Our tractographic and radiotelemetric results provide an insight on the deterioration of baroreflex-mediated sympathetic vasomotor tone during experimental HE. As visualized by DTI, there is prominent connectivity between the NTS and RVLM under resting conditions. On the other hand, the circuit that includes caudal ventrolateral medulla (CVLM) as an intermediate between the NTS and RVLM depicted in the classical literature^33^ was rarely observed. Direct projection of barosensitive neurons in the NTS to the RVLM has been reported^37^, as is the possibility for those NTS neurons to synapse with GABAergic interneurons in the RVLM. As such, it is conceivable that the baroreflex-mediated sympathetic vasomotor tone of the rats in our study is sustained by a tonic inhibitory input from the NTS to RVLM, which in turn lessens the tonic excitatory action of these premotor sympathetic neurons on vasomotor tone. During the initial stage of experimental HE, reducing the tonic inhibitory influence of the NTS on RVLM as indicated by a reduction in the NTS-RVLM connectivity in effect increases the outflows from the RVLM to the blood vessels, leading to our observed augmentation of the baroreflex-mediated sympathetic vasomotor tone that effectively sustains BP. Intriguingly, the significant disruption of the connectivity between the NTS and RVLM as reflected by the progressive reduction of the power density of the BLF component in the SBP spectrum signifies the gradual impairment of this component of the baroreflex. The ultimate severance of this NTS-RVLM connectivity and disappearance of the BLF power that signify the occurrence of defunct baroreflex-mediated sympathetic vasomotor tone suggest that brain death, which precedes cardiac death, has ensued during the final stage of HE.

Our tractographic and radiotelemetric results also revealed that the modus operandi of cardiac vagal baroreflex during experimental HE is discernibly different. Under resting conditions, the cardiac vagal baroreflex is sustained by a tonic excitatory input from the NTS to NA, which in turn exerts a tonic inhibitory action on the heart, known classically as the vagal brake. During the initial stage of experimental HE, our results showed that by increasing the tonic excitatory influence of the NTS on NA that enhances the inhibitory actions of the vagus nerve on the heart (reinforcement of vagal brake), the cardiac vagal baroreflex is effectively augmented to sustain HR. Intriguingly, with the progressively reduced but maintained NTS-NA connectivity during the progression of experimental HE, the functionality of cardiac vagal baroreflex is retarded but sustained, leading to the preservation of HR until the abrupt occurrence of cardiac death.

Results from DWI in the present study provided further mechanistic insights into the high mortality associated with HE. DWI is a MRI technique that measures the relative motion of water across cell membrane^38^. Unlike vasogenic edema that accompanies the breakdown of blood-brain-barrier, cytotoxic edema represents simply the redistribution of water from extracellular to intracellular compartments. Without a change in local constituents, this phenomenon is not evident under T_1_- or T_2_-weighted imaging. The only MRI sequence that is able to identify cytotoxic edema is DWI. As cells swell because of inward shift of water, there is a commensurate decrease in diffusion, identified as low signal on ADC. A decrease in ADC values in the forebrain of patients with fulminant hepatic failure has been reported^29^,^30^. Cytotoxic edema is also reported in forebrain structures in a rat model of acute liver failure^28^. Our DWI data extended those observations from the forebrain to provide the first demonstration that cytotoxic edema in the brain stem also occurs during experimental HE. Importantly, the significant reduction in λ_‖_ and the insignificant changes in λ_**⊥**_ measured in the brain stem during experimental HE further ascertained that the disrupted NTS-RVLM or NTS-NA connectivity revealed by our tractographic analysis is restricted to water movements in the dorsal-ventral direction, the trajectory of the fiber tracks that connect the NTS and NA or the NTS and RVLM. It has been reported that whereas axonal injury is associated with a loss of λ_‖_^39^, demyelination leads to an increase in λ_**⊥**_^40^. It follows that our observed reduction in λ_‖_ and insignificant changes in λ_**⊥**_ suggest that axonal injury may also underlie the disrupted NTS-RVLM or NTS-NA connectivity. This suggestion, however, awaits further delineation.

Our immunofluorescence and biochemical results suggested that the observed cytotoxic edema may be related to elevation of tissue ammonia and swelling of astrocytes in the NTS. Hyperammonemia and astrocytic swelling have been related to cerebral edema in patients with cirrhosis^30^,^41^,^42^. As the major route of ammonia detoxification in brain, astrocyte converts ammonia to glutamine via glutamine synthase. Glutamine is subsequently transported to mitochondria and is hydrolyzed by phosphate-activated glutaminase located in the mitochondrial membrane, yielding glutamate and ammonia. Albrecht and Norenberg^43^ suggest that it is this glutamine-derived ammonia within the mitochondria that brings about astrocyte dysfunction, including cell swelling. Of note is that methionine sulfoximine, an inhibitor of glutamine synthase, reduces ammonia-induced astrocyte swelling and cerebral edema^44^,^45^.

Results from our tractographic, biochemical and pharmacological experiments further suggest that nitrosative stress in the form of upregulated peroxynitrite in the NTS or RVLM is causally related to the impairment of the baroreflex during experimental HE. This notion is in line with the report that oxidative/nitrosative stress in the cerebral cortex is associated with HE in patients with cirrhosis^7^. Our observed elevation in tissue ammonia and swelling of astrocytes in the NTS or RVLM is again of interest. Augmented NO concentration and NOS activity because of increased and effective recycling of citrulline to arginine in brain structures, including the brain stem, has been reported in rats subjected to acute ammonia toxicity^46^. In addition, the high level of glutamine-derived ammonia within mitochondria reportedly brings about generation of ROS/RNS or cerebral RNA oxidation^7^,^43^. It should be pointed out that previous work from our laboratory^16^,^17^,^18^,^19^,^20^,^21^,^22^,^23^,^24^,^25^ emphasizes that it is nitrosative stress induced by peroxynitrite in key nuclei of the baroreflex circuit, rather than oxidative stress, that underpins defunct baroreflex-medicated sympathetic vasomotor tone, leading to brain death. This notion is again substantiated in the present study. We found that pretreatment with an active peroxynitrite decomposition catalyst. but not an antioxidant, protected against the reduction in SBP or HR, and impairment of baroreflex-medicated sympathetic vasomotor tone or cardiac vagal baroreflex during experimental HE. Since on intracisternal infusion, FeTMPyP will reach both the NTS and RVLM, it is likely that its anti-nitrosative effects at the NTS will be manifested by reversing the disrupted NTS-RVLM or NTS-NA connectivity and decreased FA. At the same time, FeTMPyP may antagonize the nitrosative stress in the RVLM by blunting the retarded power density of the BLF component of the SBP spectrum during experimental HE via an anti-apoptotic action^17^,^20^.

We are cognisant that an obvious limitation to our results and interpretations is the unpredictability of the time of death in our animals. As such, we can only resort to using the averaged values of the MRI/DWI/DTI or cardiovascular parameters collected daily at 11:00–14:00 for statistical analyses. While allowing us to assess parallel trends, this practice offers only mean information on group data. Another similar limitation is that to achieve optimal imaging results, the time taken for each DWI or DTI scan was approximately 30 min. This implies that the ADC, FA or number of fiber tracts values obtained in effect represent the averaged changes during this time-window while the radiotelemetric parameters were undergoing waxing or waning fluctuations.

Previous clinical studies from our laboratory showed that the disappearance of baroreflex-mediated sympathetic vasomotor tone signifies brain death in patients who succumbed to various etiologies^12^,^13^,^14^,^15^. It follows that brain death because of nitrosative stress-induced loss of connectivity between the NTS and RVLM, accompanied by nitrosative stress-induced apoptotic cell death in the RVLM^17^,^20^, offers a reasonable modus operandi for the high mortality associated with HE. Our findings may therefore be extended to the clinic to aid in the design of alternative therapies against this fatal eventuality. Liver transplantation is currently the only remedy for patients with HE. However, enough exceptions to the rule that HE is reversible by liver transplantation have been reported^47^,^48^. Given the universal shortage of donors, this is most undesirable. The harsh reality is that failure of a patient with HE to regain consciousness after liver transplantation implies one donor liver squandered. As such, our findings may also be extended to the clinic as a prognostic predictor of successful reversal of HE after liver transplantation. Specifically, steps may be taken to determine the threshold of impairment of baroreflex-mediated sympathetic vasomotor tone based on the power density of the BLF component of SBP signals and connectivity between NTS and RVLM (FA values or number of fiber tracts) below which liver transplantation will likely fail to elicit its remedial actions in the recipient. This threshold, should it be identified, will be an invaluable prognostic tool in our evaluation of the suitability of patients with HE for liver transplantation.

## Methods

### Ethics statement

All experimental procedures carried out in this study were approved by the Institutional Animal Care and Use Committee of the Kaohsiung Chang Gung Memorial Hospital, and were in compliance with the guidelines for animal care and use set forth by that Committee.

### Rat model of hepatic encephalopathy

As recommended by the International Society for Hepatic Encephalopathy and Nitrogen Metabolism Commission^49^, a TAA-induced acute liver failure model was generated, using male adult Sprague-Dawley rats (278 ± 28 g, n = 172). In brief, rats received intraperitoneal injection of TAA (Sigma-Aldrich, St. Louis, MO, USA) at doses of 300, 400 or 500 mg kg^−1^ in saline at 11:00 for 3 consecutive days; controls received saline instead. The progressive severity of experimental HE over time was graded daily at 09:00–11:00 according to Zimmerman *et al*.^33^ with modifications^35^.

### Blood pressure and heart rate recording by radiotelemetry and determination of spontaneous baroreflex

Similar to our previous studies^31^,^50^, changes in SBP and HR of the rats were recorded by radiotelemetry under a conscious state in their home cages. The transmitted BP signals were digitized and processed based on feature extraction to characterize the BP cycles. Continuous, on-line and real-time spectral analysis of SBP signals based on fast Fourier transform was used to detect temporal fluctuations of the BLF component (0.25–0.8 Hz in rat) in the SBP spectrum. We demonstrated previously^36^ that the power density of this spectral component is a valid index for baroreflex-mediated sympathetic vasomotor tone. BRS determined by on-line detection of spontaneous sequences of consecutive increases or decreases in SBP associated with parallel changes in HR was used as the index for cardiac vagal baroreflex^31^. Concurrent 24-h changes in SBP, HR, power density of BLF band, BRS and activity of the animals were continuously recorded before and at least for 4 days after the first injection of TAA. The averaged values of those parameters at 11:00–14:00 were used for statistical analyses.

### MRI, DWI and DTI

We carried out sequential MRI acquisition in rats anesthetized with isoflurane at 11:00–14:00 using a 9.4 T horizontal-bore animal MR scanning system (Biospec 94/20, Bruker, Ettingen, Germany) before and at least for 4 days after the first injection of TAA. As a routine, we first recorded T_2_-weighted sagittal anatomical images to provide the landmark structures (Fig. S3A) for orientation to perform T_2_-weighted coronal anatomical reference imaging (Fig. S3B) on 11 adjacent slices from a restricted area of the brain stem that covered the medullary portion of the NTS, NA, RVLM and CVLM.

DWI was acquired using spin echo-DtiEpi sequence on the same spatial dimension as in the T_2_-weighted coronal reference imaging. ParaVision 5.1 software (Bruker) and MIstar (ver. 3.2.63; Apollo Medical Imaging Technology, Melbourne, Australia) were applied to process the DWI and ADC maps respectively (Table S5). Multiple b values were used to obtain more accurate diffusion value.

Also employing identical spatial dimension as in the T_2_-weighted coronal reference imaging, we evaluated the connectivity between NTS and NA, key brain stem nuclei in the cardiac vagal baroreflex circuit, and between NTS and RVLM or CVLM, key brain stem nuclei in the baroreflex-mediated sympathetic vasomotor tone circuit, using the spin echo-planar imaging-DTI sequence in the same coronal plane (Fig. S3C; Table S6). Post-processing of the image data entailed an analysis of fiber tractography and determination of DTI indices, using the National Taiwan University DSI studio (http://dsi-studio.labsolver.org). Bilateral fiber tracts between the NTS and NA, or between the NTS and RVLM, were selected for tractographic evaluation. In addition, regions-of-interest (ROI)-based analysis was performed to quantify two DTI indices, FA and number of fiber tracts^51^. In brief, based on the anatomical locations of the NTS, NA and RVLM in the T_2_-weighted coronal images (Fig. S3B) and making reference to a rat stereotaxic atlas^52^, two ROIs were marked manually on corresponding areas in the FA map of the brain stem (Fig. S3C). The ROI dimensions for NTS, NA and RVLM were respectively 3 × 6, 2 × 2 and 2 × 4 pixels (Fig. S3C). Values of FA, which ranged from 0 (isotropy) to 1 (maximum anisotropy), were derived from the standard deviation of the three eigenvalues (λ_1_, λ _2_, λ_3_) of the diffusion ellipse of probability density function. To calculate fiber numbers, the tracts that passed through both of NTS and NA or NTS and RVLM were counted using the streamline tracking method.

### Intracisternal infusion of test agent by osmotic minipump

Intracisternal infusion of FeTMPyP (Calbiochem, San Diego, CA, USA), an active peroxynitrite decomposition catalyst^53^, or tempol (Calbiochem), an antioxidant^54^, was delivered for at least 6 days by an osmotic minipump (Alzet 2001; Alzet Corp, Cupertino, CA, USA)^50^. Control infusion of aCSF served as the volume and vehicle control.

### Immunofluorescence staining and biochemical analyses

Immunofluorescence staining of the brain stem against GFAP antiserum was performed. ImageJ program was used to quantify GFAP immunoreactivity in regions of interest. Tissue samples obtained by micropunch bilaterally from the NTS or RVLM were subject to various biochemical assays. The level of ROS/RNS was measured by electron spin resonance using 1-hydroxy-3-carboxyl-2,2,5,5-tetramethyl-pyrrolidine (CPH; Enzo; Lausen, Switzerland) and 0.2 mM diethylenetriaminepentaacetic acid (DTPA; Sigma-Aldrich, St. Louis, MO, USA) as the trapping agents or by a ROS/RNS assay kit (Cell Biolabs, San Diego, CA, USA)^55^. Protein level of NOS II or nitrotyrosine was determined by Western Blot analysis^17^. The amount of protein expression was expressed as the ratio relative to β-actin protein.

### Blood Chemistry

Blood samples (0.5 ml) for measurement of AST, ALT, T-Bilirubin, albumin, or ammonia (NH_3_) were taken before and after TAA administration. The blood levels of NH_3_ and serum levels of AST, ALT, T-Bilirubin, or albumin were determined by an autoanalyzer (Fujifilm, Tokyo, Japan).

### Histological Examination

The liver was removed immediately after sacrifice and fixed in 10% neutral-buffered formalin for 24 h. The tissues were dehydrated, embedded in paraffin, sectioned at 4 μm thickness, and stained by hematoxylin and eosin for histopathological analysis.

### Ammonia ELISA

We measured ammonia in the NTS or RVLM tissue lysates by an ammonia colorimetric assay kit (BioVision, Milpitas, CA, USA) according to the manufacturer’s protocol. After the colorimetric reaction, the optical density was read at 570 nm in a micro plate reader (Thermo, Vantaa, Finland).

### Statistical analysis

All values are expressed as means ± SEM. Student’s t-test, one-way or two-way analysis of variance with repeated measures was used to assess group means, as appropriate, followed by the Dunnett or Scheffé multiple-range test for post hoc assessment of the individual means. Dunnett test was used for intra-group comparison against baseline, and Scheffé test was used for inter-group comparison^56^. P < 0.05 was taken to indicate statistical significance.

## Additional Information

**How to cite this article:** Tsai, C. Y. *et al*. Nitrosative Stress-Induced Disruption of Baroreflex Neural Circuits in a Rat Model of Hepatic Encephalopathy: A DTI Study. *Sci. Rep.* **7**, 40111; doi: 10.1038/srep40111 (2017).

**Publisher's note:** Springer Nature remains neutral with regard to jurisdictional claims in published maps and institutional affiliations.

## Supplementary Material

Supplementary Information
