# Supplementary material for: Nitrosative Stress-Induced Disruption of Baroreflex Neural Circuits in a Rat Model of Hepatic Encephalopathy: A DTI Study
Source: Sci Rep. 2017 Jan 12;7:40111. doi: 10.1038/srep40111 (PMC5228038; doi:10.1038/srep40111)
Supplement: Supplementary Information [file srep40111-s1.pdf]

Supplementary Information

Nitrosative Stress-Induced Disruption of Baroreflex Neural Circuits in a Rat Model of Hepatic Encephalopathy: A DTI Study

Ching-Yi Tsai, Chia-Hao Su, Julie Y.H. Chan & Samuel H.H. Chan

Institute for Translational Research in Biomedicine,

Kaohsiung Chang Gung Memorial Hospital, Kaohsiung, Taiwan, Republic of China

Supplementary Figures

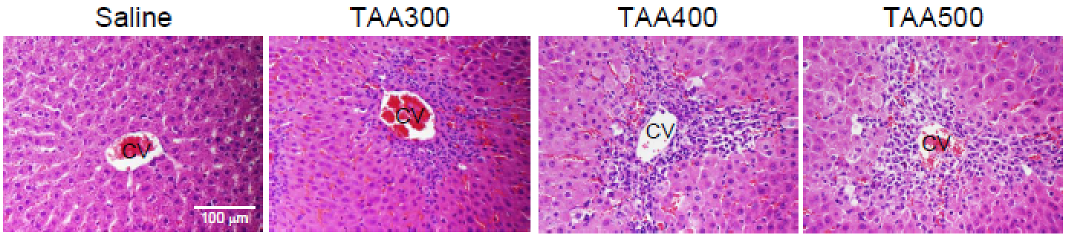

Figure S1. Photomicrographs showing typical histological changes in the liver stained with hematoxylin and eosin 4 days after animals received the first TAA administration (300, 400 or 500 mg kg^{-1}). Note dose-related increase in necrotic cells and neutrophil infiltration around the centrilobular vein (CV).

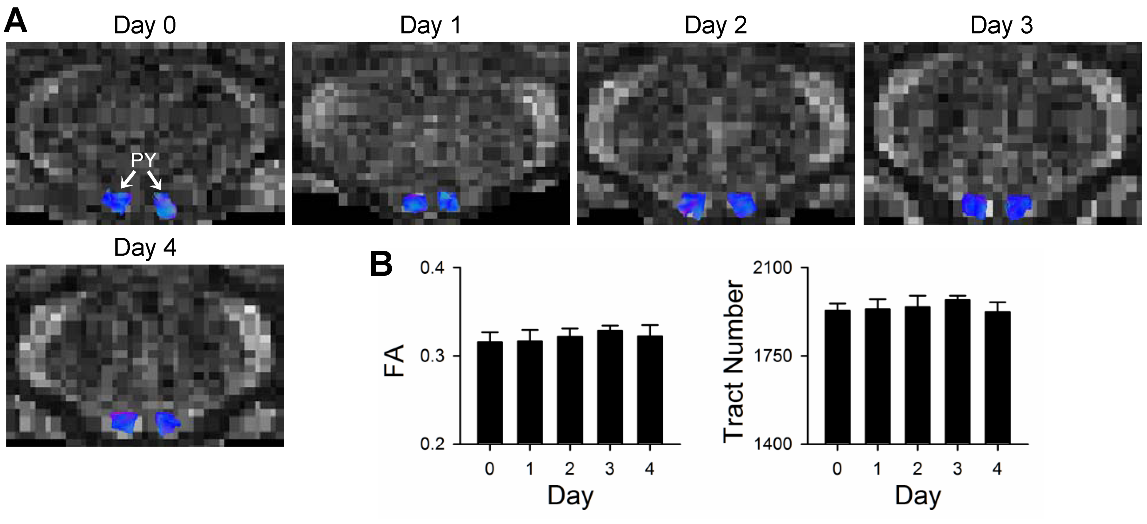

Figure S2. Lack of effects on connectivity in the pyramidal tracts during experimental HE. Illustrative example of imaging the bilateral pyramidal tracts (PY) in rats by DTI **(A)**; or the associated temporal changes in FA or number of tracts, quantitative indices for the magnitude of connectivity in DTI analysis, **(B)** before (Day 0), during (Days 1 to 3) and after (Day 4) the first TAA administration. Values in **(B)** are mean ± SEM, n = 7 animals. No statistical significance among all groups (P > 0.05) in two-way ANOVA with repeated measures. Note consistent presence of caudal-rostral connectivity coded in blue, and the lack of dorsal-ventral connectivity coded in green.

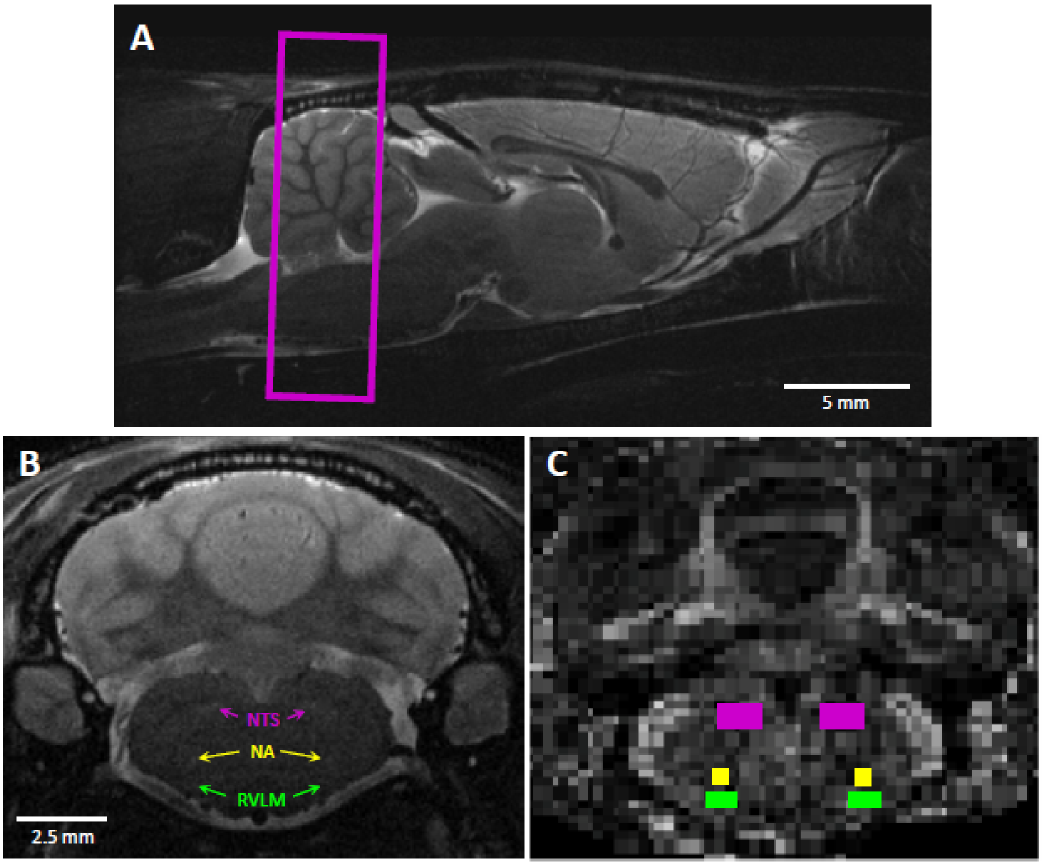

Figure S3. Procedures for magnetic resonance imaging (MRI) and tractography with diffusion tensor imaging (DTI) of rat brain stem. **(A)** High resolution T_2-weighted sagittal anatomical reference image showing demarcation of the area of the brain stem (rectangular box) that was subjected to tractographic analysis with DTI. **(B)** T_2-weighted coronal anatomical reference image showing detailed locations of the bilateral nucleus tractus solitarii (NTS), nucleus ambiguus (NA) and rostral ventrolateral medulla (RVLM). **(C)** Regions of interest (ROIs) marked manually on both sides of the fractional anisotropy (FA) map of the brain stem, which correspond to the locations of bilateral NTS, NA and RVLM in the T_2-weighted coronal image in **(B)**, to be used to quantify the DTI indices.

Supplementary Tables

Table S1. Blood chemistry in rats after administration of TAA (300, 400 or 500 mg kg^{-1}).

	**AST (U/l)**		**ALT (U/l)**		**T-Bilirubin (mg/dl)**		**Albumin (g/dl)**		**Ammonia (µg/dl)**	
	Day 0	Day 4	Day 0	Day 4	Day 0	Day 4	Day 0	Day 4	Day 0	Day 4
Saline	68 ± 3	65 ± 3	30 ± 2	29 ± 2	0.40 ± 0.06	0.36 ± 0.04	3.83 ± 0.10	3.88 ± 0.08	264 ± 42	230 ± 48
TAA300	67 ± 4	833 ± 130*†	32 ± 4	362 ± 66*†	0.37 ± 0.06	0.99 ± 0.22*†	3.79 ± 0.08	3.21 ± 0.06*†	239 ± 48	550 ± 120*†
TAA400	67 ± 4	1058 ± 265*†	29 ± 1	433 ± 85*†	0.37 ± 0.07	2.03 ± 0.45*†	3.89 ± 0.10	3.20 ± 0.18*†	242 ± 43	781 ± 106*†
TAA500	69 ± 4	1126 ± 253*†	30 ± 3	426 ± 100*†	0.41 ± 0.08	2.17 ± 0.56*†	3.85 ± 0.10	3.10 ± 0.15*†	236 ± 42	753 ± 158*†

Values are mean ± SEM of 6-8 animals per group. *P < 0.05 versus corresponding baseline (Day 0) group in the post hoc Dunnett multiple-range analysis; and †P < 0.05 versus saline group on Day 4 in the post hoc Scheffé multiple-range analysis. AST, aspartate aminotransferase; ALT, alanine aminotransferase; T-Bilirubin, total-bilirubin.

Table S2. Clinical scores of rats treated with TAA.

Group	Day 0	Day 1	Day 2	Day 3	Day 4
Saline	0	0	0	0	0
TAA300	0	0	0.86 ± 0.10	1.76 ± 0.10	2.19 ± 0.15
TAA400	0	0	1.12 ± 0.12	1.82 ± 0.10	2.50 ± 0.41
TAA500	0	0	1.59 ± 0.12	2.06 ± 0.09	2.88 ± 0.54

Animals received 3 consecutive daily doses of TAA (300, 400 or 500 mg kg^{-1}). Scores were taken before the injection of TAA. Values are mean ± SEM, n = 16-18 animals per group.

Table S3. Mortality rate at Day 4 in experimental HE.

Group	Total Number	Death Number	Death Rate
Saline	20	0	0 %
TAA300	24	2	8.3 %
TAA400	21	10	47.6 %
TAA500	22	14	63.6 %

Table S4. Axial diffusivity ($\lambda_{||}$) and radial diffusivity ($\lambda_{\perp}$) values in the brain stem of rats after administration of TAA.

	Whole brain stem	
	$\lambda_{\|\|}$ (x10^{-4} mm^2/s)	$\lambda_{\perp}$ (x10^{-4} mm^2/s)
Day 0	9.97 ± 0.05	6.43 ± 0.13
Day 1	9.70 ± 0.31	6.42 ± 0.28
Day 2	9.42 ± 0.35	6.28 ± 0.26
Day 3	9.00 ± 0.15*	6.13 ± 0.14
Day 4	8.90 ± 0.12*	6.06 ± 0.16

Values are mean ± SEM, n = 5-7 animals per group. *P < 0.05 versus baseline (Day 0) group in the in the post hoc Dunnett multiple-range analysis.

Table S5. Parameters for diffusion weighted imaging (DWI) between the nucleus tractus solitarii (NTS), nucleus ambiguus (NA) and rostral and caudal ventrolateral medulla (RVLM, CVLM) of the rat, using spin echo-DtiEpi sequence in the coronal plane covering the eleven 400-µm slices in the T_2-weighted coronal reference images without gap.

**Parameter**	
Field of view	20 mm × 20 mm
Matrix dimension	128 × 128 pixels
Spatial resolution	156 µm × 156 µm
Slice thickness	400 µm
Interslice distance	400 µm
Echo time	27 ms
Repetition time	3500 ms
b value/direction	100, 300, 500, 700, 800, 1000 s/mm^2
Number of direction	1
Number of b0 images	1
Gradient duration	2.5 ms
Gradient separation	8 ms
Number of segments	2
Number of averages	36
Acquisition time	29 min 24 s

Table S6. Parameters for diffusion tensor imaging (DTI) between the NTS, NA and RVLM of CVLM of the rat, using spin echo-planar imaging-DTI sequence in the coronal plane covering the eleven 400-μm slices in the T_2-weighted coronal reference images without gap.

**Parameter**	
Field of view	20 mm × 20 mm
Matrix dimension	96 × 96 pixels
Spatial resolution	208 μm × 208 μm
Slice thickness	400 μm
Interslice distance	400 μm
Echo time	19.5 ms
Repetition time	4500 ms
Number of diffusion directions	46
Optimized b value/direction	1500 s/mm^2
Number of b0 images	5
Gradient duration	2.7 ms
Gradient separation	10 ms
Number of averages	4
Acquisition time	30 min 36 s

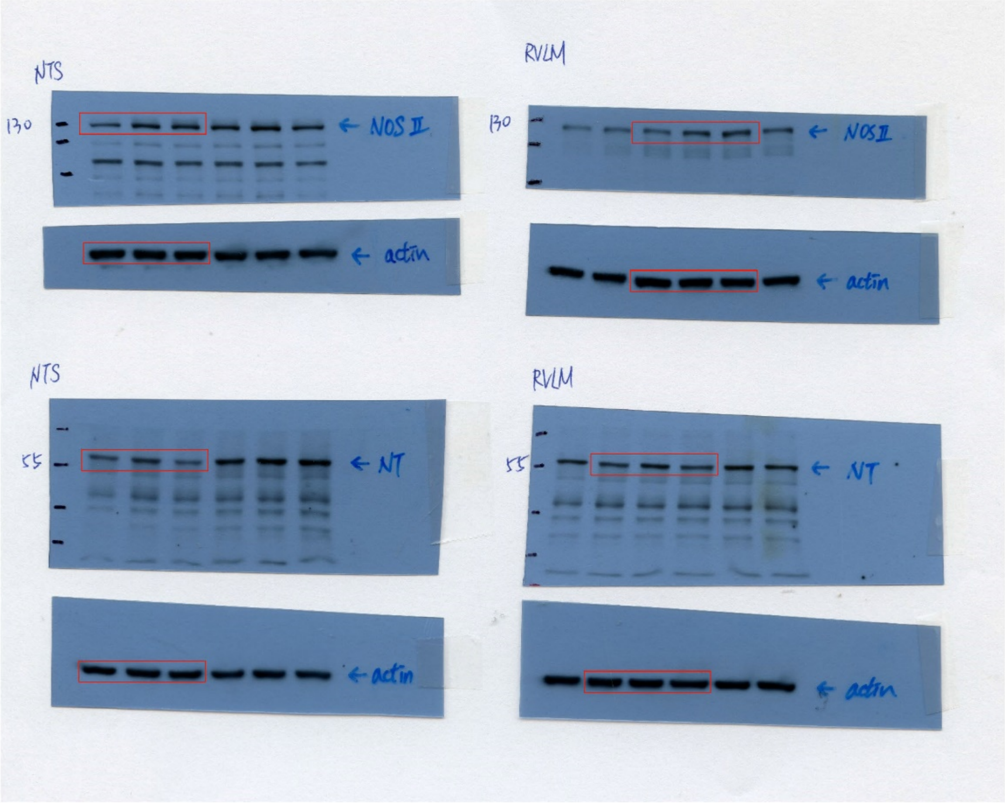

Full-length western blots in conjunction with Fig. 6C.
